# A novel, rapid technique for clearing leaf tissues

**DOI:** 10.1002/aps3.11391

**Published:** 2020-09-30

**Authors:** Emmanuel García‐Gutiérrez, Fernando Ortega‐Escalona, Guillermo Angeles

**Affiliations:** ^1^ Facultad de Biología Universidad Veracruzana Campus Xalapa Veracruz Mexico; ^2^ Red de Ecologia Funcional, Instituto de Ecología, A.C. (INECOL) Xalapa, Veracruz Mexico

**Keywords:** diaphanization, leaf clearing, leaf epidermis, leaf venation, stomata, trichomes

## Abstract

**Premise:**

Clearing leaves is a highly useful practice for many taxonomic, ecological, physiological, and eco‐physiological aspects of research. Using traditional methods, the procedure for clearing a leaf (referred to as diaphanization) can take several days or even weeks. In our laboratory we developed a technique, originally used for dissociating wood, that yields excellent epidermal and leaf venation preparations clearly showing the details of epidermal cells, hydathodes, trichomes, leaf margins, and leaf venation, in a maximum of three days.

**Methods and Results:**

A solution originally used to macerate wood (Franklin’s solution) was used to accelerate the process of clearing leaves. Using this approach, it is possible to obtain clear images of the leaf adaxial and abaxial epidermal surfaces and leaf venation. Our technique works with both fragile and sturdy leaves, as well as with thin roots and stems. A few examples are presented.

**Conclusions:**

Franklin’s solution is very simple to prepare and easy to manipulate. This new technique has the advantage of separating the leaf epidermal layers and venations, which can then be clearly observed.

The observation of leaf epidermal and venation details is important for many disciplines. Leaf functional traits are affected by climate change and could be used to predict ecosystem compositions in the coming decades (Brodribb et al., 2010; Soudzilovskaia et al., 2013; Bickford, 2016). The distribution and abundance of stomata and trichomes (epidermal hairs) on the leaf surface can reveal useful information about the habitat in which a plant grows (Meinzer and Goldstein, 1985; Meinzer et al., 1985; Soudzilovskaia et al., 2013; Blackman et al., 2018). This information is crucial for making deductions about both extant and historical environments, with paleobotanists relying on what they can observe on petrified leaf surfaces (Brodribb et al., 2010). On the other hand, another character used in taxonomic studies (Sen and Hennipman, 1981; Roth‐Nebelsick et al., 2001; Tejero‐Díez et al., 2010; Kolivand et al., 2019), leaf venation, is directly related to the hydraulic architecture of plants (Sack et al., 2012; Blackman et al., 2018). Stomatal densities (i.e., the number of stomata per square millimeter of leaf surface) and stomatal indices (i.e., the number of stomata as a percentage of the total number of epidermal cells) provide useful tools for plant ecologists studying species plasticity, and help to determine which characteristics are expected for species occupying new ecological niches (Sack et al., 2012; Sack and Buckley, 2016; Bertolino et al., 2019). For example, species growing in artificially enriched CO_2_ atmospheres tend to have higher stomatal indices (Konrad et al., 2017).

Several leaf clearing techniques have been widely used for many years (Table 1). Most of these methods can be grouped in one of the following categories: (1) tissue degradation using organic solvents, (2) tissue clearing using aqueous solutions, (3) hyperhydration of the tissue, or (4) the embedding of tissue into hydrogel (for a detailed description of this classification, see Richardson and Lichtman, 2015; Ariel, 2017). The technique described here falls into the first category. It employs Franklin’s solution (Franklin, 1945; Miksche and Berlyn, 1976), which has traditionally been used to macerate angiosperm wood, and uses two common reagents (i.e., glacial acetic acid and 35% hydrogen peroxide [hydrogen peroxide can be obtained in various degrees of purity, from 30% to 49.9%]) to obtain a clear view of both epidermal surfaces, separated from the leaf venation, which can then be stained appropriately and mounted on slides. In this manner, the leaf epidermis can be clearly observed without the visual distraction of the leaf venation underneath, and the leaf venation can be observed without obstruction by the epidermal cells.

**Table 1:** Comparison of different techniques for clearing leaves.

Technique	Advantages	Disadvantages	References
Tissue degradation using organic solvents	Good quality Fairly fast (several days)	Does not allow immune staining or 3D imaging Sample needs to be cut Sample tends to be very fragile Can damage the microscope lens	Richardson and Lichtman, 2015; Ariel, 2017
Tissue clearing using aqueous solutions	Supports immune staining and 3D imaging Applies to whole organs	Extremely slow (weeks to months) Samples tend to become opaque or have low quality	Richardson and Lichtman, 2015; Ariel, 2017
Hyperhydration of the tissue	Good quality Supports immune staining and 3D imaging Applies to whole organs	Extremely slow (weeks to months)	Richardson and Lichtman, 2015; Ariel, 2017
Embedding in hydrogel	Good quality Can support immune staining and 3D imaging Applies to whole organs	Extremely slow (weeks to months)	Richardson and Lichtman, 2015; Ariel, 2017
Modified Franklin’s solution	Good quality Somewhat fast (2–3 days) Can treat full organs	Samples can become fragile or may not be fully digested if time and temperature are not adequately adjusted. Might not be useful for 3D imaging or immune staining	This work

## METHODS AND RESULTS

### Overview of the leaf clearing method

This technique can be used with fresh, fixed (any fixative), or dried leaves (i.e., herbarium samples). Appendix 1 provides a summary of the procedure and the equipment used to make observations and capture images. Appendix 2 presents flowcharts that guide the reader through the choice of which processing steps to use for different tissues and provide options for preserving diaphanized samples. For dry leaves, it is necessary to boil the samples in tap water for 20–30 min. After cooling, the tissue samples are transferred to Franklin’s solution (equal volumes 35% hydrogen peroxide and glacial acetic acid). Fixed leaves must first be washed to remove the fixative, then transferred to Franklin’s solution. Fresh leaves only need to be washed with running tap water to remove dust and debris. In all cases, samples can be cut to the desired size. It is advisable to divide large leaves transversely in the middle or cut along one leaf margin to have a place from which to extract the leaf venation.

**FIGURE A1:**
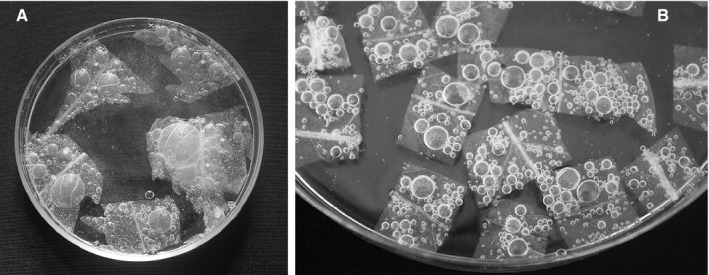
The tissue digestion process. (A) Leaf segments in a 100 × 15‐mm glass Petri dish, in Franklin’s solution. After 2 h in this solution, large gas bubbles appear trapped between the leaf two epidermises. (B) A different sample at higher magnification, showing a closer view of the bubbles. The leaf venation is visible.

Whole leaves or leaf segments are partially digested with Franklin’s solution at room temperature for up to 24 h, depending on leaf thickness. For coarser leaves, digestion can be assisted by heat (60°C) or by extending the digestion time in the Franklin’s solution. This digests the parenchyma tissue, leaving the epidermis and leaf veins intact. If required, after washing the cleared leaf with water, leaf samples can be placed in a weak solution of sodium hypochlorite (25–50% in water) to make the epidermis transparent. The chemical reactions produced in the mesophyll cells in contact with the Franklin’s solution create air bubbles that help to separate the abaxial and adaxial epidermises (see Fig. A1 in Appendix 1), loosening the leaf veins. The samples are then washed very carefully with several changes of tap water to remove any remaining Franklin’s solution or sodium hypochlorite. If desired, samples can then be stained with a 0.01% safranin aqueous solution or with 0.05% aqueous toluidine blue O. Safranin is a dye generally used by plant anatomists to stain lignin in cell walls, nuclear material, and the cytoplasm. Toluidine blue O is a polychromatic stain that, when used at an appropriate pH (slightly basic), stains cell walls a greenish blue color, nuclei deep purple, and cytoplasm pink. After removing the excess stain, either with water or 50% ethanol, the leaf veins are pulled out of the envelope formed by the two epidermises and treated separately in individual Petri dishes.

At this point, one must opt for permanent or temporary slides. For permanent slides, the samples are transferred from absolute ethanol to mixtures of absolute ethanol : methyl salicylate in 2 : 1, 1 : 1, and 1 : 2 (by volume) ratios, for 10 min in each combination. The samples are then transferred to pure methyl salicylate. Three to five drops of synthetic resin (see details of the resin we used in Appendix 1) are placed in the center of a microscope slide. The sample should be placed on top of the resin drops, with a few more resin drops applied on top of the sample. The cover slip is placed over the sample, taking care not to introduce air into the resin. The sample is then dried on a slide warmer for at least 24 h.

**FIGURE 1:**
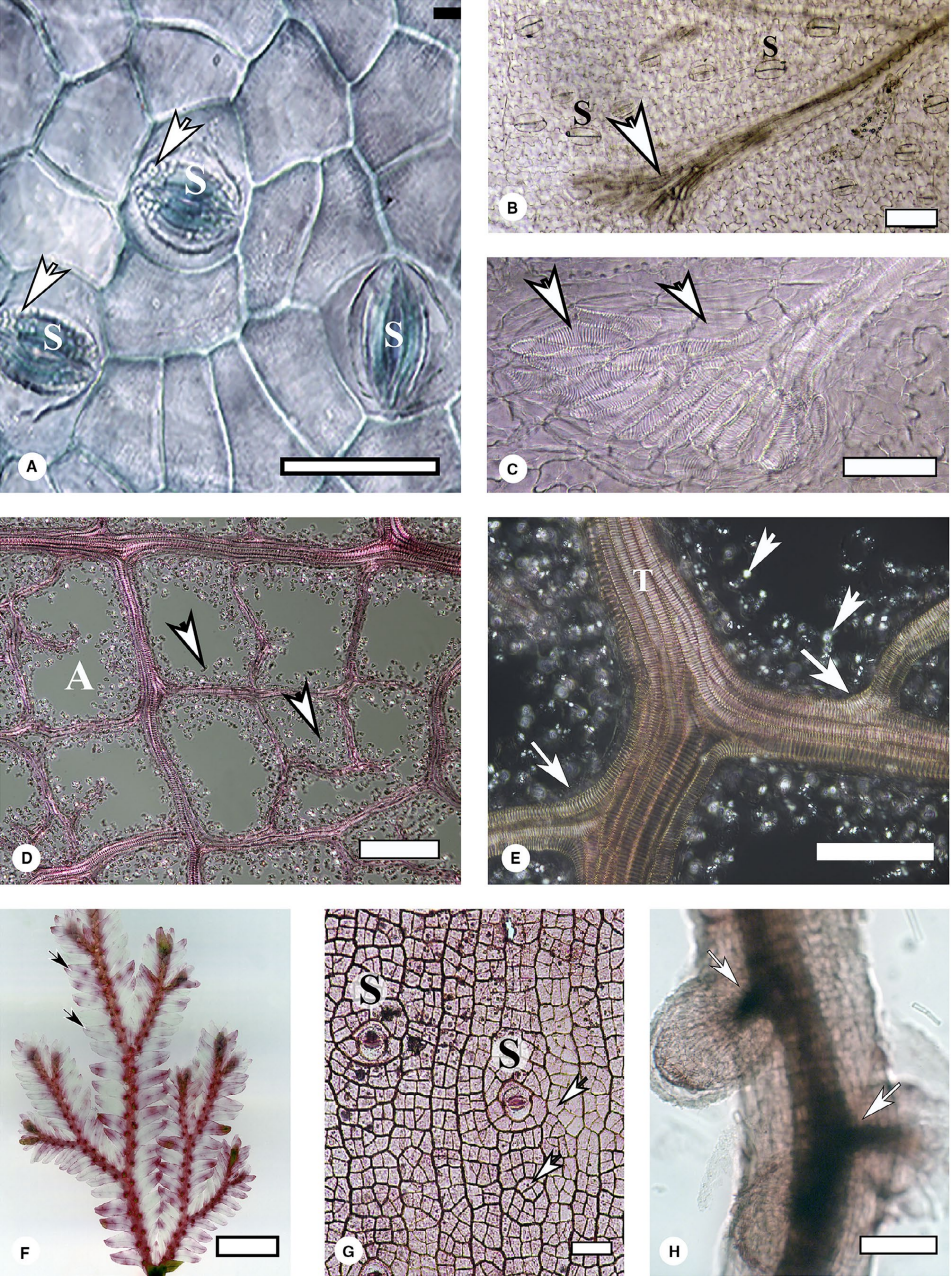
Examples of plant tissues cleared using our method. (A) Adaxial leaf epidermis of *Psittacanthus schiedeanus*, after being separated from the veins and mesophyll. The sample was stained with toluidine blue O, mounted in glycerol, and observed with an inverted microscope in bright‐field mode. Three paracytic stomatal apparatuses (S) are visible. Some plastids can be seen in the occlusion cells (arrows). Scale bar = 100 µm. (B) Cleared and unstained *Blechnum appendiculatum* leaf. All cell contents were digested after 16 h of treatment. The whole leaf was mounted in synthetic resin and photographed under polarized light. A minor vein ends in a hydathode (arrow), just beneath the sorum. Some stomata (S) are observed on the abaxial epidermis. Scale bar = 100 µm. (C) An enlargement of (B), showing details of the tracheids (arrows) forming the hydathode. Scale bar = 50 µm. (D) A portion of the venation of a *Piper hispidum* leaf, separated from the mesophyll and epidermis. Tracheids are lined up by the cytoplasm remnants and starch grains, seen as bright dots (arrows) against the dark background; this image was taken under polarized light. An areole (A) is visible as a closed loop formed by the tracheids. Scale bar = 100 µm. (E) Magnification of (D). Franklin’s solution did not dissociate a thick vein formed by seven tracheids (T). Bright dots (arrowheads) are starch grains and small crystals shown by polarized light. Vein branchings (large arrows) can also be observed. Scale bar = 100 µm. (F) *Selaginella* sp. cleared for 4 h at room temperature in Franklin’s solution and stained with 0.01% aqueous safranin. Leaf veins are clearly visible under bright‐field microscopy after staining (arrows). Scale bar = 2.5 mm. (G) Epidermis of *Rhipsalis baccifera* isolated from an adult plant by digestion with Franklin’s solution for 6 h at room temperature. The epidermal cells (arrows), of uniform dimensions, form a distinctive pattern. Some stomata are visible (S). Scale bar = 100 µm. (H) Mycorrhizic root of *Pinus hartwegii*, cleared with Franklin’s solution for 4 h at 60°C and stained with 0.01% aqueous safranin. The stele shows lateral connections with two lateral roots (arrows). Scale bar = 100 µm.

For temporary slides, pure glycerol is used as a substitute for resin. Samples are taken from the Petri dish in glycerol and are placed on a glass slide. A cover slip is placed on top of the sample, taking care to avoid causing the glycerol to overflow. Pictures can be taken immediately. Another option is to place the sample in a glass‐bottomed tissue culture dish with glycerol for direct observations with an inverted microscope (Fig. 1A shows an example of this procedure). Once the leaf veins or epidermises are mounted as described above, they can be observed under a microscope with the desired optics. Several examples are shown in Fig. 1.

Using the technique described in this paper, the tissue may be easily broken after the bleaching, and some starch may not be fully digested. These situations can be reduced or eliminated by adjusting the time and temperature used for the clearing.

### Testing the leaf clearing method

We tested our technique using leaves of different textures and thickness, as well as some other organs (roots and thin stems), selected from plants growing in our locality (Xalapa, Veracruz, Mexico).

#### Successful preparation of diverse leaf types

Figure 1A shows an example of an isolated adaxial epidermis from the hemiparasite *Psittacanthus schiedeanus* (Cham. & Schltdl.) G. Don (Loranthaceae). It was stained with a 0.05% solution of toluidine blue O, washed in water, gradually dehydrated up to absolute ethanol (increasing steps of 25%, 50%, 75%, and 100% ethanol), and transferred to a glass‐bottomed tissue culture dish in glycerol. It was observed with an inverted microscope, using bright‐field imaging. Some plastids can be observed in the occlusive cells of the stomata. Figures 1B and 1C depict cleared young leaves of *Blechnum appendiculatum* Willd., demonstrating that the technique works well for thin laminae. The samples were digested for 16 h at room temperature, dehydrated, cleared with methyl salicylate, and mounted in synthetic resin without staining. The images were taken with a compound microscope, under polarized light. Stomata and a hydathode plus the wavy margins of epidermal cells can be simultaneously observed under low magnification (Fig. 1B). At higher magnifications, the details of the helical thickenings of the tracheids forming the hydathode can be observed (Fig. 1C). Figures 1D and 1E show the isolated veins of a *Piper hispidum* Sw. leaf. Tracheids are lined up by cytoplasm remnants and starch grains, seen as bright dots against a dark background in this image taken under polarized light. They remained attached to the tracheids even after the samples were shaken for 15 min in an ultrasound bath. No further digestion with more aggressive reagents (like sodium hydroxide) was attempted. The lateral veins can be seen to be connected to the main vein (Fig. 1E). For very fragile tissues, such as in *Selaginella* P. Beauv. (Fig. 1F), the Franklin’s solution does not need to be heated. For these tissues, digestion was carried out at room temperature for 4 h, after which the cleared leaves were stained with aqueous 0.1% safranin solution, dehydrated in ethanol, cleared with methyl salicylate, and mounted with synthetic resin.

#### Successful preparation of other tissue types

Figure 1G shows a portion of epidermis isolated from an adult stem of *Rhipsalis baccifera* (Sol.) Stearn, an epiphyte cactus that does not develop a periderm. The epidermal cells are of similar size, forming an even reticular pattern. Two stomata are visible. Roots can also be cleared with our technique, as illustrated in Fig. 1H. In this example, the digestion of a *Pinus hartwegii* Lindl. root was carried out at 60°C for 2 h. After cooling at room temperature, the roots were washed, stained with a 0.1% aqueous solution of safranin, washed again, dehydrated in ethanol, cleared with methyl salicylate, and mounted in synthetic resin. The image was taken at low magnification using a dissecting microscope.

In Table 1, we present a synthesis of different techniques for clearing tissues, providing a comparison of their applications as well as the advantages and disadvantages of each method.

## CONCLUSIONS

This technique offers a quick and effective way to clear leaves and other plant organs. The protocol is especially useful for investigating the epidermis and veins, which can be treated jointly or separately. Our technique has not been successful for observing the venation of succulent leaves, however. The two main advantages of this technique are that glacial acetic acid and hydrogen peroxide are inexpensive and readily available in laboratories, and that it reduces the time needed for the diaphanization of a leaf. The minor disadvantages of this technique are outweighed by the low cost, speed, and utility of this protocol.

## APPENDIX 1 Detailed summary of the leaf clearing protocol.

### Equipment

- Model MZ8 dissecting microscope (Leica Microsystems, Wetzlar, Germany)
- Eclipse E600 compound microscope (Nikon, Tokyo, Japan)
- EOS Rebel T3i/EOS 600D digital camera (8 MP; Canon, Tokyo, Japan)
- DMI6000B inverted microscope (Leica Microsystems), used in bright‐field mode, aided with a DFC450C digital color camera CCD (5 MP; Leica Microsystems)

NOTE: Use glassware throughout the process. Use nitrile gloves and eye protection, and work under a hood if possible.

### Clearing tissue

*Starting from dry leaves:*

1. Boil dry leaves in tap water for 20–30 min.
2. Let them cool.
3. Transfer to Franklin’s solution (equal volumes of 35% H_2_O_2_ and glacial acetic acid at room temperature).

*Starting from fresh or fixed leaves:*

1. Wash under the faucet, to remove dust from the surfaces.
2. Wash abundantly with water (fixed leaves only).
3. Transfer to Franklin’s solution at room temperature for 2 h, or until the samples turn whitish translucent. If a faster process is desired, place the sample in solution in an oven at 50–60°C.
4. When large gas bubbles appear trapped between the two epidermises (Fig. A1), it is an indication that veins can be removed.
5. Wash abundantly with tap water.
6. OPTIONAL: If samples are not satisfactorily clean of debris, transfer to a weak (25–50%) solution of sodium hypochlorite for 15–20 min and wash abundantly with tap water.
7. Stain with aqueous or ethanolic dye for 5–10 min (e.g., 0.1% aqueous safranin, 0.05% toluidine blue O, or another dye of your preference).
8. Remove the excess stain by washing with water or 50% ethanol.
9. Observe under a dissecting microscope.
10. Carefully pull out the veins with forceps, assisted with a fine camelhair brush.
11. From this point, process the epidermises and veins separately, in parallel.

### Mounting slides

*Permanent slides:*

1. Dehydrate with increasing concentrations of ethanol (50%, 70%, 96%, and 100%) for 2 min at each step.
2. Place the specimen in methyl salicylate (a.k.a. wintergreen oil).
3. Transfer to a 1 : 1 mixture of methyl salicylate and synthetic resin (we used a 60% synthetic resin dissolved in xylenes; Golden Bell Reactivos Materiales y Abastos Especializados, S.A. de C.V., Mexico City, Mexico) for 1 h, well covered to avoid contact with air.
4. Put some drops of the synthetic resin in the center of a microscope slide.
5. Carefully place the sample in the synthetic resin without trapping air bubbles.
6. Apply some more resin on top of the specimen.
7. Place the coverslip over the specimen.
8. Dry the slide using a slide warmer for at least 24 h.

*Temporary slides*:

1. Transfer to equal volumes of glycerol and 100% ethanol (for 2 min or longer).
2. Transfer to pure glycerol. Leave overnight.
3. Mount with glycerol for temporary preparations.

OPTIONAL: Transfer samples to glass‐bottomed tissue culture disks, if you prefer to observe them through an inverted microscope.
